# Creative Approaches to Global Cancer Research and Control

**DOI:** 10.1200/GO.20.00237

**Published:** 2020-07-27

**Authors:** Anne F. Rositch, Christopher Loffredo, Maria T. Bourlon, Paul C. Pearlman, Clement Adebamowo

**Affiliations:** ^1^ Department of Epidemiology, Johns Hopkins Bloomberg School of Public Health, Baltimore, MD; ^2^ Department of Oncology, Lombardi Comprehensive Cancer Center, Georgetown University, Washington, DC; ^3^ Hemato-Oncology Department, Instituto Nacional de Ciencias Médicas y Nutrición Salvador Zubirán, Mexico City, Mexico; ^4^ National Cancer Institute Center for Global Health, Rockville, MD; ^5^ Institute of Human Virology, Department of Epidemiology and Public Health, Marlene and Stewart Greenebaum Comprehensive Cancer Center, University of Maryland School of Medicine, Baltimore, MD; ^6^ Institute of Human Virology, Abuja, Nigeria; ^7^ Center for Bioethics and Research, Ibadan, Nigeria

## Introduction

Cancer is a significant source of morbidity and mortality globally, but the burden is greater in low- and middle-income countries (LMICs), which together account for > 70% of the nearly 20 million annual incident cancers. Increasing life expectancy, advancements in human development, and the adoption of a westernized lifestyle are contributing to a rapidly growing cancer epidemic in LMICs.^1-4^ These trends highlight the urgency and importance of developing “Creative Approaches to Global Cancer Research and Control,” the theme of the 8th Annual Symposium on Global Cancer Research (ASGCR). This meeting was scheduled to convene on April 17, 2020, in Washington, DC, but had to be postponed because of the SARS-CoV2 pandemic—a potent reminder of the need for and the value of a global approach to health and diseases, including cancer.

Creative approaches from individual researchers and collaborative and transdisciplinary teams, complemented by cancer societies and both governmental and nongovernmental organizations, are needed to move the global oncology field forward. Such approaches include innovative study designs, methods, and technologies plus improvements in training and curricula and expanded platforms for networking and virtual interactions that take into account the realities of high-, middle-, and low-resource settings.^5-7^ Many of these approaches, which we planned to discuss at the ASGCR, have the potential to narrow the cancer disparities between high-income countries and LMICs by lowering costs and improving access or quality of screening, early detection, diagnosis, and treatment.

The 8th ASGCR steering committee, which comprises leaders from National Cancer Institute (NCI)–accredited cancers centers in the Baltimore-Washington area, ASCO, the American Association of Cancer Research, and NCI, along with researchers and practitioners who registered for the meeting are a paradigmatic example of multiple stakeholder engagement. Such engagement is required to brainstorm on the research, implementation and dissemination, and methods and technologies that either are available or need to be developed for successful global cancer control. Together, innovation and collaboration will be essential in pushing forward an agenda to recognize and combat the global burden of cancer.

## Current Research on Creative Approaches

### Technological approaches.

Prevention, early detection, and treatment depend on a range of technologies, some of which are not currently used in LMICs because of cost, limited medical and public infrastructure, and trained personnel. Emerging point-of-care technologies (POCTs) promise to revolutionize care delivery in such settings. Attractive features of POCTs include lower cost, increased durability, and more streamlined approaches that are useful to providers who practice outside traditional clinical settings.^8^ Advances in engineering and test chemistry have enabled the detection of sophisticated biomarkers on small, portable platforms that require minimal training to use. These advances have led to high-quality home-based testing or self-testing, which extends the impact from the clinic into the community.^9^

Because 95% of the global population is covered by mobile networks, mobile health (mhealth) continues to be one of the most promising avenues for providing access to health services.^10^ In parallel, the emergence of mHealth 2.0, including connectivity to wearables and the Internet of things, has led to a glut of new health data in high-resource settings.^11^ This creates unique research opportunities and challenges for LMICs, where gaps persist in availability of these technologies, their infrastructural requirements, and personnel needed to implement them. Looking forward, increased broadband Internet coverage will further enable telementoring, telemedicine, and telepathology, while 5G networks promise a reduction in latency that could enable real-time educational, research, and clinical interventions from halfway around the world.

The speed and capability of computers continue to grow, and this has driven innovative applications of machine learning and artificial intelligence (MLAI) for development of high-quality computer-assisted cancer detection and diagnosis that can be implemented with little training. Nevertheless, significant implementation barriers exist in LMICs, including a lack of reliable local data to train and validate AI systems, a limited trained workforce, and the lack of regulatory capacity to oversee and manage MLAI.^12^ These barriers notwithstanding, progress has been made in the use of MLAI in medical physics, where researchers are developing MLAI tools to automate dose planning for radiation treatment, and in cervical cancer, where deep learning shows some initial promise for revolutionizing screening in community settings.^13,14^

### Methodological approaches.

While much attention has been paid to novel technologies such as AI, nanotechnology, and omics, the development of creative approaches to their evaluation and integration into existing cancer prevention and research strategies is equally, if not more, important.^15-19^ These methodologies include the collection of a broad range of lifestyle data, including comprehensive daily physical activities, dietary intakes, environmental exposures, and physiologic parameters, through wearable and mHealth technologies; deployment of novel clinical trial designs; and wider adoption of observational epidemiology methods that engage big data, bioinformatics, and biostatistics for integrative epidemiology, systems epidemiology, and phenome-wide association studies.^20-35^ Innovations in the development and use of open source tools and rapidly growing social and economic infrastructure will support implementation of these innovative research and management programs that can inform local cancer prevention and management efforts.^36-41^ Engagement of novel implementation and dissemination science toolkits and multidisciplinary collaborations that engage the best team science methods have great potential to rapidly translate new knowledge into cancer public health policies and practice.^42-48^

### Training, infrastructure, and funding approaches.

Formal changes to oncology curricula to include global oncology are needed to enable the development of necessary skills. Furthermore, there is need for new or expanded networks of physicians, scientists, and institutions that would foster development, research, implementation, and communication of successful creative approaches globally. In-person and virtual meetings to foster understanding of resources settings can enable the development and deployment of effective solutions regardless of the resource levels and the creation of global inventories of successful global cancer control and treatment strategies.^49,50^

Funding opportunities have increased worldwide for global oncology over the past few decades, and cancer societies have played an important role in garnering support for expanded funding. Several grant mechanisms now exist that are dedicated to this area of research. As stated in a recent National Institutes of Health study, there has been a substantial increase in global oncology research and training conducted by NCI-designated cancer centers, and much of this work is happening in LMICs.^51^ This progress has created a structured funding pathway for global oncology specialists and will pave the way for the development of new and more-effective strategies to improve equitable access to cancer control and treatment in all resource settings. In contrast, there is limited funding to support LMICs to scale up successful cancer control and treatment interventions similar to the President’s Emergency Program for AIDS Relief or the Global Fund to Fight AIDS, Tuberculosis and Malaria. Efforts to integrate HIV and noncommunicable disease treatment and prevention programs have met with limited success. Resolution of these challenges is bound to rise to the top of the global oncology agenda going forward.

In conclusion, these are exciting and challenging times for global oncology. Never before has such a range of tools—from enabling technologies to novel study designs and big data approaches—been available to bring to bear on the problems of cancer prevention and control. The SARS-CoV2 pandemic raises new challenges for the global oncology community to develop innovative approaches to collaboration, communication, and implementation virtually and effectively over long distances. Our past successes in global oncology have been recognized in previous conferences, whereas the present conference offered an opportunity to tell new and compelling stories that highlight progress in the global effort to reduce the burden of cervical cancer, the development and implementation of new educational curricula in LMICs, and innovative approaches to improve quality and access to radiotherapy in LMICs, to name but a few examples. It is our hope that the global oncology community will come together to resolutely face the post–COVID-19 world and continue to develop creative and innovative approaches to global cancer control, treatment, and research. Our field is poised to emerge stronger than ever and renewed in our resolve to foster increasing equity across the globe. Our future conferences will disseminate the progress we will make.
